# Autoantibodies predate the onset of systemic lupus erythematosus in northern Sweden

**DOI:** 10.1186/ar3258

**Published:** 2011-02-22

**Authors:** Catharina Eriksson, Heidi Kokkonen, Martin Johansson, Göran Hallmans, Göran Wadell, Solbritt Rantapää-Dahlqvist

**Affiliations:** 1 Department of Clinical Immunology, Umeå University, SE-901 85 Umeå, Sweden; 2 Department of Public Health and Clinical Medicine/Rheumatology, Umeå University, SE-901 85 Umeå, Sweden; 3 Department of Nutritional Research, Umeå University, SE-901 85 Umeå, Sweden; 4 Department of Virology, Umeå University, SE-901 85 Umeå, Sweden

## Abstract

**Introduction:**

Autoantibodies have a central role in systemic lupus erythematosus (SLE). The presence of autoantibodies preceding disease onset by years has been reported both in patients with SLE and in those with rheumatoid arthritis, suggesting a gradual development of these diseases. Therefore, we sought to identify autoantibodies in a northern European population predating the onset of symptoms of SLE and their relationship to presenting symptoms.

**Methods:**

The register of patients fulfilling the American College of Rheumatology criteria for SLE and with a given date of the onset of symptoms was coanalysed with the register of the Medical Biobank, Umeå, Sweden. Thirty-eight patients were identified as having donated blood samples prior to symptom onset. A nested case-control study (1:4) was performed with 152 age- and sex-matched controls identified from within the Medical Biobank register (Umeå, Sweden). Antibodies against anti-Sjögren's syndrome antigen A (Ro/SSA; 52 and 60 kDa), anti-Sjögren's syndrome antigen B, anti-Smith antibody, ribonucleoprotein, scleroderma, anti-histidyl-tRNA synthetase antibody, double-stranded DNA (dsDNA), centromere protein B and histones were analysed using the AtheNA Multi-Lyte ANA II Plus Test System on a Bio-Plex Array Reader (Luminex^200^). Antinuclear antibodies test II (ANA II) results were analysed using indirect immunofluorescence on human epidermal 2 cells at a sample dilution of 1:100.

**Results:**

Autoantibodies against nuclear antigens were detected a mean (±SD) of 5.6 ± 4.7 years before the onset of symptoms and 8.7 ± 5.6 years before diagnosis in 63% of the individuals who subsequently developed SLE. The sensitivity (45.7%) was highest for ANA II, with a specificity of 95%, followed by anti-dsDNA and anti-Ro/SSA antibodies, both with sensitivities of 20.0% at specificities of 98.7% and 97.4%, respectively. The odds ratios (ORs) for predicting disease were 18.13 for anti-dsDNA (95% confidence interval (95% CI), 3.58 to 91.84) and 11.5 (95% CI, 4.54 to 28.87) for ANA. Anti-Ro/SSA antibodies appeared first at a mean of 6.6 ± 2.5 years prior to symptom onset. The mean number of autoantibodies in prediseased individuals was 1.4, and after disease onset it was 3.1 (*P *< 0.0005). The time predating disease was shorter and the number of autoantibodies was greater in those individuals with serositis as a presenting symptom in comparison to those with arthritis and skin manifestations as the presenting symptoms.

**Conclusions:**

Autoantibodies against nuclear antigens were detected in individuals who developed SLE several years before the onset of symptoms and diagnosis. The most sensitive autoantibodies were ANA, Ro/SSA and dsDNA, with the highest predictive OR being for anti-dsDNA antibodies. The first autoantibodies detected were anti-Ro/SSA.

## Introduction

Systemic lupus erythematosus (SLE) is a heterogeneous disease with diverse clinical manifestations and variable severity in individual patients and between different patient populations [1,2]. A typical pathophysiological sign in SLE patients is the production of autoantibodies directed against nuclear antigens, which precede the development of clinical manifestations [3,4]. In particular, antibodies against double-stranded DNA (anti-dsDNA) have been shown to increase just prior to a diagnosis of SLE [5]. Individuals who develop SLE have also been found to gradually fulfill the clinical classification criteria that are preceded by the appearance of associated autoantibodies before diagnosis [6]. Furthermore, in patients defined as having undifferentiated connective tissue disease, a diagnosis of SLE was predicted in a 5-year follow-up study on the basis of the presence of anti-dsDNA antibodies [7].

There are several autoimmune diseases that are recognised by exhibiting a long preclinical phase during which susceptible individuals who later develop disease can be identified by the presence of autoantibodies [8-11]. The development of a rheumatic disease in asymptomatic mothers expressing anti-Sjögren's syndrome antigen A (Ro/SSA) and/or anti-Sjögren's syndrome antigen B (La/SSB) antibodies, and identified by the birth of a child with a congenital heart block, was found to be relatively common at 48% [12]. In another study, the detection of anti-La/SSB antibodies predated clinical evidence of Sjögren's syndrome by months and in some cases by years [13]. Furthermore, in an animal model of SLE, mice immunized with human Ro/SSA developed autoimmunity not only towards this molecule but also towards other immunologically similar molecules in a process equivalent to epitope spreading [14].

The presence of antinuclear antibodies (ANAs) was shown to predate the development of SLE in a small study conducted in Finland [15]. In the study by Arbuckle *et al. *[3], the frequency of producing at least one SLE-related autoantibody years before diagnosis was high at 88%. ANAs were present in 78% of the cases, anti-dsDNA antibodies were present in 55% and anti-Ro/SSA antibodies were present in 47%. Furthermore, the appearance of these antibodies appeared to follow a predictable course [3]. Anticardiolipin antibodies have been found to precede both the diagnosis of SLE and the development of clinical manifestations of thrombosis by a number of years [16].

The aim of this study was to analyse, using multiplex technology, the autoantibodies predating the onset of symptoms of SLE in individuals in a patient population in northern Europe and to relate these autoantibodies to the first recorded symptom of disease.

## Materials and methods

### Patients and controls

The register of patients with SLE attending the Department of Rheumatology, University Hospital, Umeå, Sweden, with a known date of the onset of symptoms was coanalysed with the registers of the Medical Biobank (Umeå, Sweden) and of the maternity cohort (that is, the record of samples obtained for rubella screening of pregnant women) from northern Sweden. All SLE patients had been evaluated clinically. A total of 38 patients (3 male and 35 female, of whom 37 fulfilled four and one fulfilled only three of the American College of Rheumatology (ACR) criteria for SLE [17,18]) were identified as having donated blood before the onset of any symptoms of disease. One of the patients also fulfilled the criteria for mixed connective tissue disease [19]. Nineteen of the patients were identified from the Medical Biobank (on the basis of plasma withdrawal), and the other 19 were identified from among the maternity cohort collection (on the basis of sera withdrawal). All individuals in the county of Västerbotten are continuously invited to donate blood samples to the Medical Biobank, the plasma from which is stored at -80°C in a biorepository, and blood samples are drawn from all pregnant women with the sera stored at -20°C. Full details of the conditions for recruitment and the collection and storage of blood samples have been described previously [10].

A nested 1:4 case-control study was undertaken with the 38 identified individuals, referred to hereinafter as "presymptomatic" individuals, and randomly selected controls (*n *= 152) from the same population-based cohorts matched for sex, age and date of blood sampling as well as area of residence. The mean age at the time blood sampling of the individuals who subsequently developed SLE was 36.9 years (age range, 16.8 to 60.2 years) and that of the matched controls was 36.7 years (age range, 17.8 to 62.3 years). The patients' ages at the time of sampling, the time predating the onset of symptoms and diagnosis and the time after sampling until the onset of symptoms are presented in Table 1 for both the Medical Biobank (stratified by sex) and maternity cohorts.

**Table 1:** Age at sampling, at onset of symptoms and disease onset and predating time presented as median values (Q1, Q3)

	Medical Biobank		
Patient characteristics	Females (*n *= 16)	Males (*n *= 3)	Maternity cohort (*n *= 19)
Median age at sampling	50.1 (40.2, 52.3)	50.2 (49.2, 60.1)	24.7 (21.7, 29.0)
Median age at symptom onset	52.0 (46.8, 61.2)	52.3 (51.0, 62.1)	31.7 (26.5,39.1)
Median age at diagnosis	53.5 (48.0, 62.7)	52.8 (51.1, 63.1)	37.8 (30.2, 43.1)
Predating time between sampling and symptom onset	5.28 (1.44, 7.88)	2.03 (1.74, 2.06)	6.74 (3.0, 9.24)

Samples from three prepatients and six controls, all from the maternity cohort, were no longer available; that is, insufficient sera were available for analysis. The frequencies of nonsmokers, ex-smokers and current smokers among the presymptomatic patients were 47.2%, 26.3% and 26.3%, respectively. The equivalent data are not available for the controls.

The study was approved by the Regional Ethics Committee of the University Hospital in Umeå, and all participants gave their written informed consent.

### Analysis of autoantibodies

Autoantibodies against Ro/SSA (52 and 60 kDa), La/SSB, dsDNA, ribonucleoprotein (RNP), Smith (Sm), histidyl-tRNA synthetase (Jo-1), scleroderma (Scl-70), centromere protein B and histones in plasma from 19 presymptomatic individuals and matched controls (*n *= 76) (Medical Biobank), in sera from 16 presymptomatic individuals and matched controls (*n *= 76) (maternity cohort) and in sera from SLE patients (n = 38) were collected during the disease. All autoantibodies were detected using the multiplex AtheNA Multi-Lyte ANA II Plus Test (Zeuss Scientific, Raritan, NJ, USA) and analysed on a Bio-Plex Array Reader (Luminex^200 ^Labmap™ system; Luminex Corp., Austin, TX, USA). The cutoff level for a positive value for each autoantibody recommended by the manufacturer was used, that is, 120 AU/ml for all analytes. Analyses of ANAs were performed by indirect immunofluorescence on human epidermal cell 2 (HEp-2 cells) slides (Immunoconcept, Sacramento, CA, USA) using 1:100 diluted samples. Analyses of the autoantibodies (ANAs, anti-dsDNA, anti-Ro/SSA, anti-La/SSB, anti-Sm, anti-RNP, anti-Jo-1, anti-Scl-70, anti-centromere protein B and antihistone) in the sera of the patients at diagnosis were also undertaken by the routine clinical immunology laboratory at the University Hospital. ANAs were analysed by indirect immunofluorescence with HEp-2 cells or rat tissue (in house), anti-dsDNA was analysed on *Crithidia luciliae*-coated slides (Immunoconcept) and the other autoantibodies were analysed either by enzyme-linked immunosorbent assay or by immunoblot assay.

### Statistics

Statistical calculations were performed using SPSS for Windows version 17.0 software (SPSS, Inc., Chicago, IL, USA). Continuous data were compared by nonparametric analyses with the Wilcoxon signed-rank test for matched pairs (prepatients versus SLE patients) and conditional logistic regression analyses (prepatients versus controls). The relationships between categorical data (positive versus negative) were compared using χ^2 ^analysis or Fisher's exact test as appropriate. All *P *values are two-sided, and *P *≤ 0.05 was considered statistical significant. *P *values corrected for the number of comparisons made outside the hypothesis are presented as *P *corrected (*P*_c_).

## Results

### Analyses in presymptomatic individuals and controls

Of the 35 presymptomatic individuals whose blood samples were available, 22 (63%) had any detectable autoantibodies in their blood before the onset of symptoms, that is, predating disease by a median of 4.2 years (range, 2.1 to 7.9 years). Ten of these patients expressed one autoantibody, whilst 12 others had two or more autoantibodies (range, from two to seven). The sensitivity was highest for ANAs at 45.7% with a specificity of 95%, followed by anti-dsDNA and anti-Ro/SSA antibodies, both with a sensitivity of 20% but with specificities of 98.7% and 97.4%, respectively (Table 2). The sensitivities for the other autoantibodies were between 14.3% and 2.9% at 98% to 100% specificity levels (Table 2). The odds ratio (ORs) for predicting the development of SLE were highest for anti-dsDNA at 18.13 (95% confidence interval (95% CI), 3.58 to 91.84), followed by ANAs at 11.5 (95% CI, 4.54 to 28.87) and anti-Ro/SSA antibodies at 8.94 (95% CI, 2.45 to 32.58). The ORs for the other antibodies were between 9.36 and 4.29, although the number of positive individuals was low, that is, up to five. The likelihood ratio (LR) was highest for anti-dsDNA antibodies at 15.38, followed by ANAs with a LR of 9.14.

**Table 2:** Sensitivity and specificity of autoantibodies before onset of disease symptoms in individuals who later developed SLE^a^

	Presymptomatic individuals						
Autoantibodies	Sensitivity, *n *(%)	Specificity *n *%	Controls, *n *(%)	OR	95% CI	*P *value^b^	LR
ANA	16 (45.7)***	95.0	10 (6.7)	11.5	4.54 to 28.87	<0.0001	9.14
dsDNA	7 (20.0)***	98.7	2 (1.4)	18.13	3.58 to 91.84	<0.0001	15.38
Ro/SSA	7 (20.0)***	97.4	4 (2.7)	8.94	2.45 to 32.58	<0.0001	5.56
Histone	5 (14.3)**	98.0	3 (2.0)	8.06	1.83 to 35.54	<0.001	7.15
RNP	4 (11.4)**	98.7	2 (1.4)	9.36	1.64 to 53.36	<0.01	8.77
La/SSB	3 (8.6)**	100	0				
Jo-1	3 (8.6)*	98.7	2 (1.4)	6.80	1.09 to 42.36	<0.05	6.62
Scl-70	2 (5.7)	99.3	1 (0.7)	8.85	0.78 to 100.51	ns	8.0
Sm	1 (2.9)	100	0				
Centromere protein B	(2.9)	98.7	1 (0.7)	4.29	0.26 to 70.39	ns	1.54

^a^95% CI, 95% confidence interval; ^b^*P *values were determined by using χ^2 ^test or Fisher's exact test as appropriate. . * = p < 0.05, **= p < 0.01, ***= p < 0.001

ANA, antinuclear antibody; dsDNA, double-stranded DNA; Jo-1, anti-histidyl-tRNA synthetase antibody; La/SSB, anti-Sjögren's syndrome antigen B; LR, positive likelihood value; ns, not significant; OR, odds ratio; RNP, ribonucleoprotein; Ro/SSA, anti-Sjögren's syndrome antigen A; Scl-70, scleroderma 70; Sm, Smith.

**Figure 1:**
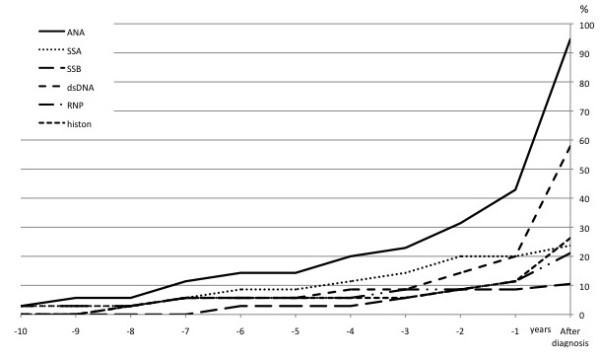
**Graph showing the accumulated number of positive individuals for each antibody**. Shown as the percentage predating disease onset in years and after diagnosis of the disease. ANA, antinuclear antibody; SSA, Sjögren's syndrome antigen A; SSB, Sjögren's syndrome antigen B; dsDNA, double-stranded DNA; RNP, ribonucleoprotein; histon, histone.

The autoantibody type to appear first before the onset of symptoms was anti-Ro/SSA antibody at a mean (±SD) of 6.6 ± 2.5 years. Anti-RNP and antihistones also appeared early at means (±SD) of 5.9 ± 2.5 years and 5.0 ± 1.5 years, respectively, although the number of positive individuals with each antibody was small, that is, four and five, respectively. The autoantibodies first detectable closest to disease onset were anti-centromere protein B at 0.2 years, anti-Sm at 0.7 years and anti-Scl-70 at a mean (±SD) of 1.4 ± 0.6 years (Table 3). The number of individuals expressing autoantibodies increased the closer they got to the onset of symptoms, that is, 12 (63%) of 19 individuals had autoantibodies present <5 years before disease onset compared with 8 (50%) of 16 individuals who had autoantibodies present ≥5 years before disease onset. The number of autoantibodies per individual also increased the closer the individual got to the onset of symptoms, particularly during the last 3 years before disease onset; however, this change did not reach statistical significance. The accumulated number of individuals who were positive for each antibody before any symptoms of disease and after disease onset is illustrated in Figure 1. In the maternity cohort, 37.5% had autoantibodies predating disease, compared with 94% in females and 100% in males from the Medical Biobank cohort.

**Table 3:** Duration in years of the various antibodies preceding the onset of symptoms and disease^a^

Antibody	Number of positive test results	Interval between positive test and onset of symptoms, mean (SD)	Interval between positive test and diagnosis, mean (SD)
Ro/SSA	7	6.6 (2.5)	8.1 (2.3)
RNP	4	5.9 (2.5)	7.5 (2.5)
Histones	5	5.0 (1.5)	6.5 (1.9)
ANA	16	4.1 (0.8)	7.5 (1.2)
La/SSB	3	4.0 (1.0)	7.0 (0.6)
dsDNA	7	3.6 (1.2)	6.6 (2.0)
Jo-1	3	2.4 (1.1)	3.1 (1.1)
Scl-70	2	1.4 (0.6)	2.1 (0.9)
Sm	1	0.7	1.1
Centromere protein B	1	0.2	6.6

ANA, antinuclear antibody; ^a^Ro/SSA, anti-Sjögren's syndrome antigen A; dsDNA, double-stranded DNA; Jo-1, anti-histidyl-tRNA synthetase antibody; La/SSB, anti-Sjögren's syndrome antigen B; RNP, ribonucleoprotein; Scl-70, scleroderma 70; SD, standard deviation; Sm, Smith.

The number of positive autoantibodies increased with age at the time of blood sampling (*P *= 0.001, Pc < 0.01). Those individuals who had autoantibodies predating disease onset were older both at the time of blood sampling and at the onset of symptoms (42.8 versus 28.3 years and 49.3 versus 36.0 years; *P *= 0.002, *P*c < 0.05, and *P *= 0.005, *P*c < 0.05, respectively). The interval between blood sampling and the onset of clinical symptoms was shorter than it was for those who had no autoantibodies in their presymptom sample; however, this finding was not statistically significantly different (mean 5.2 years versus 6.3 years before symptom onset).

### Analyses in presymptomatic individuals and at diagnosis of SLE

The mean number of autoantibodies present in predisease individuals was 1.4 and increased after disease onset to 3.1 (*P *< 0.0005). In the autoantibody positive presymptomatic individuals (*n *= 22), the mean number of autoantibodies was 2.2 before and 3.3 after a diagnosis of SLE (*P *< 0.016, *P*c < 0.1), whilst among the antibody-negative prepatients (*n *= 13), the mean number of autoantibodies after diagnosis was 2.8 (*P *< 0.002, *P*c < 0.05).

The autoantibodies present in relation to symptoms at the onset of disease are presented in Table 4. The patients with serositis (*n *= 6; four females and two of three males) at the onset of symptoms had higher frequencies of autoantibodies than did those with arthritis (*n *= 20; one of three males) and skin manifestations (*n *= 11; one male), with the mean number of autoantibodies among these patients being 2.5, 1.7 and 0.9, respectively. However, the time interval predating disease was shorter for those with primary symptoms such as serositis (median, 1.9 years) in comparison with those with arthritis (6.7 years) and skin manifestations (4.2 years). In one individual, the symptom preceding the onset of disease was nephritis without any autoantibodies detectable when analysed 3.7 years before disease onset, although at onset the patient was ANA- and anti-dsDNA-antibody-positive. There was no association between smoking and autoantibody formation in either the number of autoantibody-positive individuals or the number of autoantibodies present.

**Table 4:** Autoantibodies predating onset of SLE and presenting symptoms at disease onset^a^

Antibody	Arthritis (*n *= 19)	Skin manifestation (*n *= 11)	Serositis (*n *= 6)	Haematologic disorder (*n *= 2)	Neurologic disorder (*n *= 1)	Renal disorder (*n *= 1)
ANA	9	4	4	1	0	0
dsDNA	5	1	2	0	0	0
Ro/SSA	3	2	2	0	1	0
Histone	4	1	1	0	0	0
RNP	4	0	1	0	0	0
La/SSB	2	1	0	0	0	0
Jo-1	2	0	2	0	0	0
Scl-70	2	0	1	0	0	0
Sm	1	0	1	0	0	0
Mean number of antibodies/patient	1.6	0.9	2.5			
No antibody	7	5	2	1	0	1

^a^ANA, antinuclear antibody; dsDNA, double-stranded DNA; Jo-1, anti-histidyl-tRNA synthetase antibody; La/SSB, anti-Sjögren's syndrome antigen B; RNP, ribonucleoprotein; Ro/SSA, anti-Sjögren's syndrome antigen A; Scl-70, scleroderma 70; Sm, Smith.

In samples analysed after disease onset but during development of the disease, the concentrations of six of the autoantibodies that were positive in presymptomatic patients, namely, the autoantibodies anti-Jo-1 (*n *= 3), anti-Scl-70 (*n *= 2), anti-RNP (*n *= 2), antihistone (*n *= 2), anti-Ro/SSA (*n *= 1) and anti-centromere protein B (*n *= 1), decreased to below the cutoff values on the basis of either the multiplex detection kit or routine laboratory protocols.

## Discussion

In this study, we have shown that autoantibody seropositivity preceded the onset of SLE, as defined by ACR criteria, by years. In those individuals who subsequently developed SLE, the number of autoantibodies increased gradually. This could suggest a gradual pathogenic process over a long period. Our results are consistent with data reported in other prospective studies of asymptomatic individuals who later developed SLE [3], rheumatoid arthritis (RA) [10,11] or other autoimmune diseases [9]. ANAs were in line with the results presented by Arbuckle *et al. *[3] in that the most prevalent autoantibodies were found in individuals before the onset of symptoms. However, the frequency of the different autoantibodies predating SLE was lower in our study than the frequencies reported by others [3,20]. This could be explained by the longer time predating the onset of disease relative to the lower number of samples.

Furthermore, one must consider the ethnic background of the different patient cohorts. All of the individuals included in the present study were from northern Sweden, whilst in the two other studies cited [3,20], 62% were black in both studies, with only 29% and 26%, respectively, being of European background. Anti-extractable nuclear antigen (anti-ENA) antibodies have been found to be more common in Afro-Caribbean and African-American populations than in Caucasians [21-23]. Conversely, the importance of ethnic differences in relation to autoantibodies was not confirmed in another study [24].

Another possible explanation for the lower frequency of detectable autoantibodies in the individuals studied here is that one-half of the samples were sera from pregnant women, in whom the frequency of autoantibodies is known to generally be lower. Also, these donors were younger at the time of blood sampling, and consequently the time interval before disease onset for most of the individuals was longer. The samples from the maternity cohort were taken early in pregnancy, which can be of importance when considering that these presymptomatic individuals had a lower prevalence of autoantibodies than the remainder of the patients and also that pregnancy is, partially at least, an immunosuppressive state. These individuals were also younger at the time of the collection of blood samples, when the symptoms started and when the diagnosis of SLE was confirmed. Their samples had also been stored frozen for a longer time, which should be considered as a factor that could interfere with the analyses. After the diagnosis was established, these patients had marginally fewer autoantibodies than the other patients, although not significantly so. It has long been suggested that autoantibody formation increases with age [25,26] as was found in the present study.

In line with the other studies [3,20], anti-Ro/SSA antibodies were the first to be detected and preceded the onset of SLE by several years, whilst anti-Sm and anti-centromere protein B antibodies appeared closer to the onset of clinical symptoms. Also, as described by Arbuckle *et al. *[3], anti-dsDNA antibodies appeared at an intermediate time point. Our results differ from those of Arbuckle *et al. *in the way that ANAs appeared at an intermediate point relative to the onset of clinical symptoms and that anti-La/SSB antibodies appeared closer to the onset of symptoms. This finding is consistent with the hypothesis of a progression due to epitope spreading as previously described both in animal models and in SLE patients [27-29].

The individuals who had serositis as the first symptom had more autoantibodies and a shorter time interval between the positive blood sample and disease onset than other onset symptoms, suggesting that a more serious manifestation in the beginning of the disease is associated with faster disease development and more pronounced epitope spreading. However, we were unable to show a significant increase in the number of autoantibodies preceding symptom or disease onset, but after the onset of disease the number of antibodies increased significantly.

The OR for predicting SLE was highest for anti-dsDNA antibodies, followed by ANAs and the other autoantibodies with lower ORs, but all were within the 95% CI for the OR of anti-dsDNA antibodies. The number of individuals positive for most of the other antibodies was small: between two and five.

In this study, 6.7% of the population based controls were positive for ANAs at a preset specificity of 95%. However, ANA positivity alone in healthy individuals was not regarded as a good predictor of developing connective tissue disease [30,31]. Two controls were positive for anti-Jo-1 antibodies and one was positive for anti-Scl-70 antibodies, which are rare autoantibodies. However, because of the limited amounts of sera and plasma available from the Medical Biobank, we were not able to undertake any confirmatory analyses for anti-ENA or anti-dsDNA antibodies using alternative techniques, which would have been desirable.

The ENA and chromatin antigens are a part of all autoantigens present in the cell nuclei visualised by ANA analysis using immunofluorescence. In the nucleus, there are many antigens other than ENA or chromatin that cannot be detected by specific methods today. Comparison between multiple assays for autoantibody detection in SLE has shown variable frequency of, for example, Scl-70, with higher frequencies published using the same assay as we used in this study, suggesting a too low cutoff value, at least for Scl-70 [32,33].

In this study, we could not find any difference in autoantibody formation between smokers and nonsmokers. A significantly higher risk of dsDNA seropositivity was found in current smokers compared with those who had never smoked in a previous study of SLE patients [34]. Smoking has been suggested as an environmental factor involved in the pathogenic development of autoantibodies to citrullinated proteins and rheumatoid factor in patients with RA [35].

This study is limited by the availability of stored samples and by not having several samples collected from the same individual before the onset of symptoms. However, these individuals were patients attending one clinic, where they are followed regularly. The controls used in this study were sampled at the same time as the patients, and their samples were collected, stored and analysed in the same way.

We have also used a newly introduced multiplex technique, which is similar to that used by Heinlen *et al. *[20], thereby making comparison with the previous publication by Arbuckle *et al. *[3] more difficult. The multiplex technology is very suitable, since the amount of serum or plasma required is very small relative to the number of analytes it is possible to detect in any given sample. This is of special benefit when analysing stored serum samples from biobanks, where the volumes stored are limited.

## Conclusions

On the basis of this study, we conclude that autoantibodies against nuclear antigens can be detected several years before the onset of symptoms and SLE diagnosis in individuals who subsequently develop SLE. The highest sensitivities were for ANA, Ro/SSA and dsDNA, and anti-dsDNA antibodies had the highest predictive value for SLE. Antibodies against Ro/SSA were the first autoantibodies detected. Individuals who had serositis as the first symptom had more autoantibodies and a shorter time interval between the positive blood sample and disease onset than other onset symptoms, suggesting that more serious disease manifestation in the beginning of the disease is associated with faster disease development and more pronounced epitope spreading.

## Abbreviations

ACR: American College of Rheumatology; ANA II: antinuclear antibody test II; anti-Sm: anti-Smith antibody; dsDNA: double-stranded DNA; HEp-2: human epidermal cell 2; Jo-1: anti-histidyl-tRNA synthetase antibody; La/SSB: anti-Sjögren's syndrome antigen B; LR: likelihood ratio; OR: odds ratio; RNP: ribonucleoprotein; Ro/SSA: anti-Sjögren's syndrome antigen A; Scl-70: scleroderma 70; SLE: systemic lupus erythematosus.

## Competing interests

The authors declare that they have no competing interests.

## Authors' contributions

CE analysed and interpreted the data and was involved in drafting the manuscript. HK analysed and interpreted the data and was to some extent involved in drafting the manuscript. MJ contributed to the study design and analysed and interpreted the data. GH and GW contributed to the design of the study and were involved with the supply of the blood samples. SRD designed the study, analysed and interpreted the data and was involved in drafting the manuscript. All authors have given their final approval of the version of the manuscript to be published.
